# Effect of cross-platform gene-expression, computational methods on breast cancer subtyping in PALOMA-2 and PALLET studies

**DOI:** 10.1038/s41523-024-00658-y

**Published:** 2024-06-29

**Authors:** Maggie Chon U Cheang, Mothaffar Rimawi, Stephen Johnston, Samuel A. Jacobs, Judith Bliss, Katherine Pogue-Geile, Lucy Kilburn, Zhou Zhu, Eugene F. Schuster, Hui Xiao, Lisa Swaim, Shibing Deng, Dongrui R. Lu, Eric Gauthier, Jennifer Tursi, Dennis J. Slamon, Hope S. Rugo, Richard S. Finn, Yuan Liu

**Affiliations:** 1 The Institute of Cancer Research, Sutton, UK; 2 Baylor College of Medicine, Houston, TX USA; 3 The Institute of Cancer Research, London, UK; 4 NSABP Foundation, Pittsburgh, PA USA; 5 Pfizer Inc, La Jolla, CA USA; 6 Pfizer Srl, Milan, Italy; 7 David Geffen School of Medicine, University of California Los Angeles, Santa Monica, CA USA; 8 University of California San Francisco Helen Diller Family Comprehensive Cancer Center, San Francisco, CA USA

**Keywords:** Predictive markers, Breast cancer

## Abstract

Intrinsic breast cancer molecular subtyping (IBCMS) provides significant prognostic information for patients with breast cancer and helps determine treatment. This study compared IBCMS methods on various gene-expression platforms in PALOMA-2 and PALLET trials. PALOMA-2 tumor samples were profiled using EdgeSeq and nanostring and subtyped with AIMS, PAM50, and research-use-only (ruo)Prosigna. PALLET tumor biopsies were profiled using mRNA sequencing and subtyped with AIMS and PAM50. In PALOMA-2 (*n* = 222), a 54% agreement was observed between results from AIMS and gold-standard ruoProsigna, with AIMS assigning 67% basal-like to HER2-enriched. In PALLET (*n* = 224), a 69% agreement was observed between results from PAM50 and AIMS. Different IBCMS methods may lead to different results and could misguide treatment selection; hence, a standardized clinical PAM50 assay and computational approach should be used.

**Trial number:** NCT01740427

## Introduction

Breast cancer diagnosis and decisions regarding treatment are largely based on clinicopathologic variables such as histologic subtype, nodal status, tumor size and grade, and biomarkers such as estrogen receptor (ER) and human epidermal growth factor receptor 2 (HER2), which are suboptimal biomarkers for predicting disease outcome of targeted therapies and emerging treatments^1,2^. Using global gene-expression profiling, breast cancers can be molecularly classified into five intrinsic subtypes: luminal A (LumA), luminal B (LumB), HER2 enriched (HER2-E), basal-like (BL), and normal-like (NL)^3,4^, although these subtypes do not represent distinct disease entities but, rather, exist on a continuum. These subtypes are associated with significantly different prognoses, incidence rates between races, and survival benefits achieved from endocrine, and HER2-targeted therapies^5–9^. Molecular classification of breast cancers into intrinsic subtypes has been embraced in the medical community because of its importance for both clinical decision-making and the development of new breast cancer treatments^10^.

The PAM50 classifier, an optimally selected, minimized, 50-gene-based subtype predictor, was developed from 189 prototypical samples representing the five intrinsic subtypes to capture the major intrinsic subtypes in a general patient population in relative proportions^9,11^. The clinical value of the PAM50 classifier was validated on independent cohorts of samples^9,11^. The final PAM50 algorithm consists of centroids constructed as described by Parker et al.^9,12^. Tumor subtype classification is assigned based on the nearest of the five centroids, with distances calculated using Spearman’s rank correlation^9^. The PAM50 classifier has become the widely accepted gold standard for intrinsic subtyping. A clinical-grade, standardized version of this test is the Prosigna® Breast Cancer Prognostic Gene Signature Assay (Veracyte, Inc.), which also includes a numeric score that integrates the intrinsic subtype information with tumor size. This score has been shown to indicate the probability of cancer recurrence during the next 10 years for patients with hormone receptor-positive (HR+)/HER2-negative (HER2−) early breast cancer^13,14^.

Contemporary breast cancer trials and clinical studies are often focused on molecular subgroups as defined by HR+ and HER2+ status for inclusion criteria. However, with improving sequencing technologies, it is easier to obtain high-dimensional gene-expression data using RNAseq techniques for formalin-fixed paraffin-embedded (FFPE) samples collected from clinical trials. The publicly available PAM50 classifier algorithm and associated intrinsic subtype centroids (i.e., average profiles) were developed to capture the major intrinsic subtypes in a general population of patients with breast cancer in relative proportions. The clinicopathologic distribution of the study cohort should be carefully considered and normalized. For example, it should be determined if the study cohort has mainly ER-positive (ER+) breast cancer or triple-negative breast cancer. Furthermore, the technology platform should be calibrated for gene-expression profiling^15^. The absolute intrinsic molecular subtyping (AIMS) algorithm was originally trained to recapitulate the intrinsic subtype classification by the PAM50 algorithm and was claimed to have 77% agreement in testing^16^. This bioinformatic approach was suggested to accurately assign subtypes to individual patients regardless of normalization procedures used, relative frequencies of ER+ tumors or subtypes, or other clinicopathologic patient attributes. However, the accuracy of AIMS in predicting intrinsic subtyping by PAM50, as originally developed, has not been cross-validated by an independent study.

The aim of this analysis was to determine if there were any discrepancies between PAM50 and AIMS in regard to intrinsic subtyping by performing head-to-head comparisons of these different next-generation sequencing technologies using the same sample sets. Data were collected using tumor samples from postmenopausal women with ER+/HER2− breast cancer included in two randomized trials, PALOMA-2 and PALLET. The predictive value of the intrinsic subtypes, as assessed by the different methodological approaches, for progression-free survival (PFS) treatment benefit of palbociclib was also evaluated.

## Results

### PALOMA-2

A total of 666 patients were enrolled in PALOMA-2; of these, 455 patients had HTG gene-expression data, and 222 patients had both ruoProsigna-PAM50 and HTG-AIMS data. Baseline demographics and disease characteristics were similar between patients in the overall cohort in PALOMA-2 and those with biomarker data (Supplementary Table 1). An overall 54% agreement rate was observed between the ruoProsigna-PAM50 and HTG-AIMS methods. In total, 46% of samples (56/121) assigned as LumB subtype by the gold standard ruoProsigna-PAM50 were assigned as LumA by HTG-AIMS, and 67% (6/9) of those assigned as BL by ruoProsigna‑PAM50 were assigned as HER2-E by HTG-AIMS (Table 1). Cohen’s kappa statistic of agreement was 0.30 (*P* < 0.0001), indicating a fair agreement between the two computational subtyping methods that were generated from their respective gene-expression profiles. Since the clinical-grade Prosigna does not provide an NL subtype, we assigned the NL in AIMS to its closest subtype, LumA, to calculate a kappa statistic.

**Table 1:** Intrinsic breast cancer molecular-subtyping methods in PALOMA-2

	ruoProsigna-PAM50, *n* (%)				Total, *n*
HTG-AIMS	BL	HER2-E	LumA	LumB	
BL	**1 (11)**	0	0	0	1
HER2-E	6 (67)	**6 (30)**	6 (8)	13 (11)	31
LumA	0	2 (10)	**60 (83)**	56 (46)	118
LumB	2 (22)	12 (60)	3 (4)	**52 (43)**	69
NL	0	0	3 (4)	0	3
Total	9 (100)	20 (100)	72 (100)	121 (100)	222

Bolded numbers indicate subtype agreement between methods.

**Fig. 1:**
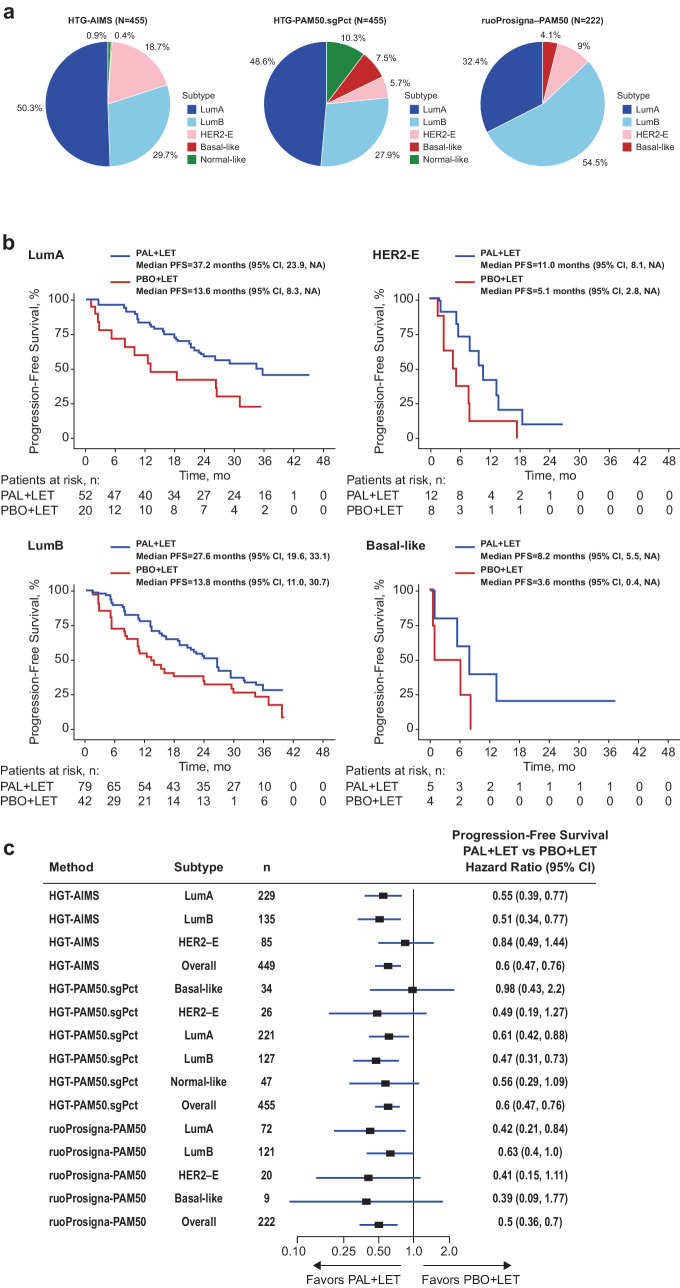
Tumor subtyping and PFS in patients from PALOMA-2. **a** Pie charts of subtype distributions by HTG-AIMs, HTG-PAM50.sgPct, and ruoProsigna-PAM50 subtyping methods among total available samples. HTG-AIMS and HTG-PAM50.sgPct were applied on the entire cohort of 455 samples; only 222 samples were available for ruoProsigna-PAM50. **b** Kaplan–Meier curves of median PFS by subtype and treatment in PALOMA-2 with ruoProsigna-PAM50, the gold standard. **c** Hazard ratios and 95% CIs for PFS by breast cancer subtype and subtyping method in PALOMA-2; for HGT-AIMS analysis, six patients were not included (four NL and two BL). *Data from Finn et al.^17^. LET letrozole, N number of patients in the analysis group, *n* number of patients, NA not available, PAL palbociclib, PBO placebo.

In PALOMA-2, the ruoProsigna-PAM50 identified 54.5% of samples (121/222) as LumB, while HTG-AIMS and HTG-PAM50.sgPct methods identified 50.3% and 48.6% of the samples as LumA, respectively (Fig. 1a). HER2-E and BL subtyping also differed between methods (ruoProsigna-PAM50, 9.0% and 4.1%; HTG-PAM50.sgPct, 5.7% and 7.5%; HTG-AIMS, 18.7% and 0.4%, respectively). Among the methods that tested for the NL subtype, HTG-PAM50.sgPct identified 10.3% as NL, whereas HTG-AIMs identified 0.9% as NL. Although the agreement between intrinsic subtyping methods was fair, the prognostic nature of the subtyping was conserved and consistent across the methods, particularly for PAM50 subtyping results on HTG and NanoString data. PFS by ruoProsigna-PAM50-derived subtype showed that palbociclib plus letrozole versus letrozole alone conferred a greater benefit for all patients regardless of LumA, LumB, HER2-E, or BL subtype; however, the sample size for the BL subtype was small, limiting the interpretation of the finding (treatment interaction *P* = 0.28; likelihood ratio test) (Fig. 1b). Hazard ratios and 95% CIs for PFS by breast cancer subtype and subtyping method are shown in Fig. 1c. These observations were corroborated by the survival analysis of HTG-PAM50.sgPct data. Regardless of the subtyping method used, palbociclib plus letrozole improved PFS compared with letrozole alone.

### Comparison of PAM50 intrinsic subtyping with AIMS on HTG data

PAM50 subtyping methods with the HTG panel on the PALOMA-2 patient samples are shown in Supplementary Table 2. As described in the Methods, proper normalization should be performed before applying the original PAM50 method on sample subgroups. As shown in Supplementary Table 2, applying the PAM50 method^9^ (without normalization), namely HTG-PAM50, tended to classify samples equally to each subtype even though PALOMA-2 included only patients with ER+/HER2− disease. After proper normalization, HTG-PAM50.sgPct improved the intrinsic subtyping assignments by reassigning many of the patients to LumA or LumB from other subtypes. HTG-AIMS classified patients into primarily LumA, LumB, and HER2-E subtypes. When comparing HTG-PAM50 and HTG-PAM50.sgPct with HTG-AIMS, the highest subtype agreement was observed with the LumA subtype (Supplementary Tables 3 and 4).

### PALLET

Molecular subtyping of PALLET samples was performed using different methods with the RNAseq gene-expression data, including RNAseq-AIMS and RNAseq-PAM50.sgMd.TC. In PALLET, 224 patients had RNAseq data at baseline, and a 69% agreement between the RNAseq-AIMS and RNAseq-PAM50.sgMd.TC computational approaches were observed. Only 4% of samples were assigned LumB by RNAseq-PAM50.sgMd.TC were assigned as LumA by RNAseq-AIMS, but 17% and 16% of samples that were assigned as LumA were classified as LumB or NL by RNAseq-AIMS, respectively (Table 2). Distributions of subtypes identified by additional subtyping methods are shown in Supplementary Table 5.

**Table 2:** Intrinsic breast cancer molecular-subtyping methods in PALLET

	RNAseq-PAM50.sgMd.TC, *n* (%)					Total, *n*
RNAseq-AIMS	BL	HER2-E	LumA	LumB	NL	
BL	3 (75)	0	0	0	1 (8)	4
HER2-E	1 (25)	3 (100)	8 (5)	6 (12)	1 (8)	19
LumA	0	0	97 (62)	2 (4)	0	99
LumB	0	0	26 (17)	41 (84)	0	67
NL	0	0	25 (16)	0	10 (83)	35
Total	4 (100)	3 (100)	156 (100)	49 (100)	12 (100)	224

**Fig. 2:**
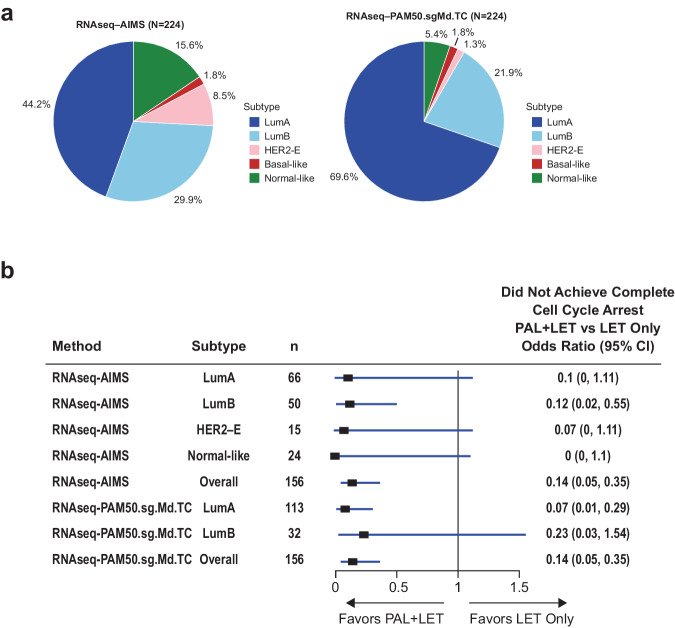
Tumor subtyping and CCCA in patients from PALLET. **a** Pie chart of subtype distributions by the RNAseq-AIMS and RNAseq-PAM50.sgMd.TC subtyping methods for PALLET samples. **b** Odds ratios and 95% CIs for non-CCCA by breast cancer RNAseq-AIMS and RNAseq-PAM50.sgMd.TC subtype in PALLET. LET letrozole, *n* number of patients in the subtype treatment group, PAL palbociclib.

In PALLET, the RNAseq-AIMS method classified 44.2% of samples as LumA, whereas RNAseq-PAM50.sgMd.TC classified 69.6% of samples as LumA, with an overall reduction in the percentage of samples classified as other subtypes (i.e., LumB, HER2-E, and NL; Fig. 2a). An equal percentage of samples was classified as BL between the two subtyping methods (1.8%). Odds ratios and 95% CIs for non-CCCA by breast cancer subtype in PALLET are shown in Fig. 2b. Overall, percentages of patients with non-CCCA were significantly lower in the palbociclib arm using both the RNAseq-AIMS and RNAseq-PAM50.sgMd.TC subtyping methods. The individual subtypes are classified with RNAseq-AIMS and PAM50.sgMd.TC also favored the palbociclib arm, which was significant for LumB patients subtyped with the RNAseq-AIMS method and LumA patients subtyped with the RNAseq-PAM50.sgMd.TC method but did not reach significance for other subtype groups owing to the small numbers of patients.

### Evaluation of the heterogeneity of intrinsic subtyping assignments

**Fig. 3:**
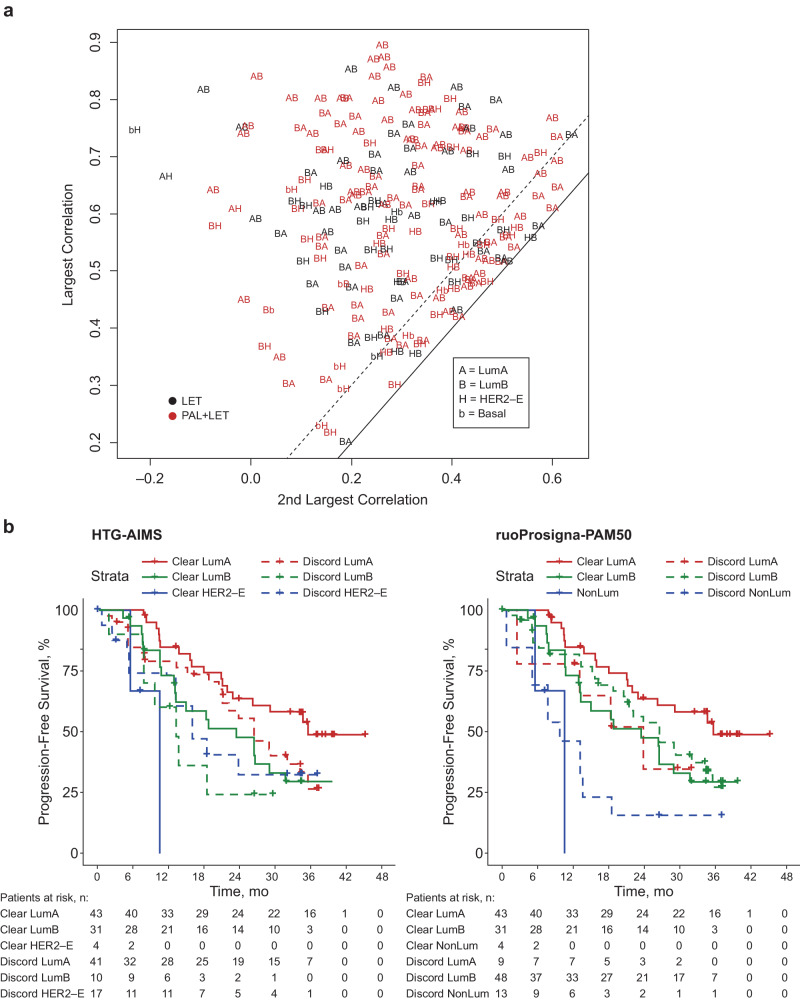
PALOMA-2 subtype correlations and PFS by subtype. **a** Plot of the largest two correlations between sample expression data and ruoProsigna subtype centroids. The solid line is the equal line, and the dashed line is 0.1 from the equal line. Each symbol is a sample. Symbol “AB” means the largest correlation coefficient is with LumA and the second largest correlation is with LumB. The same pattern follows for the remaining symbol combinations. **b** PFS in the palbociclib plus letrozole group between patients with clear-defined subtypes (solid lines) versus patients with discord subtypes (dashed lines) based on agreement between the HTG-AIMS and ruoProsigna-PAM50 methods. In the figure on the left, the subtype is based on AIMS; in the figure on the right, the subtype is based on PAM50. LET letrozole, NonLum nonluminal, PAL palbociclib.

In examining the largest and second-largest distance to PAM50 intrinsic subtyping centroids in the gold-standard ruoProsigna-PAM50 assay, the distances and correlation between the first two closest centroids were very close among some samples (Fig. 3a). Particularly, most of those close pairs were LumA‒LumB, LumB‒HER2-E, or HER2-E–BL. This suggests the potential for misclassification of samples with vague boundaries between LumA and LumB, LumB and HER2-E, and HER2-E and BL. On the other hand, it is unlikely that a LumA sample would be misclassified as HER2-E or BL, or vice versa by PAM50.

For the 222 patients in PALOMA-2 with both HTG-AIMS and ruoProsigna-PAM50 subtyping results, the subtype agreement was 54% (κ = 0.3). Patients whose subtypes agreed between the two methods were defined as subtype clear-defined (*n* = 119), and the remaining patients were subtype borderline-defined or discord. Figure 3b shows PFS among patients in the palbociclib arm with subtypes defined by HTG-AIMS (left panel) and by ruoProsigna-PAM50 (right panel). In the AIMS subtype, the clear-defined subtypes (solid lines) are highly prognostic, with LumA having better PFS than LumB, and HER2-E having the worst. However, in patients with subtype discord (dashed lines), PFS in patients with HER2-E was similar to PFS in patients with clear-defined LumB subtype. This suggests that AIMS did not separate LumB and HER2-E subtypes well, with discord HER2-E more like clear-defined LumB, and discord LumB more like clear-defined HER2-E. The same data plotted with ruoProsigna-PAM50 subtypes (Fig. 3b, right panel) showed that PFS in the discord LumA and clear-defined LumB subtypes were similar, which suggests that PAM50 separated LumA and LumB poorly, with discord LumA more like LumB and discord LumB more like LumA. The discord nonluminal subtype was very similar to clear-defined nonluminal, indicating that the ruoProsigna-PAM50‒defined nonluminal subtype was consistently assigned.

Furthermore, a numeric experiment was conducted by switching the subtype from the closest centroid to the second-closest centroid if the correlation distance was within 0.1. About 22.5% of patients (*n* = 50/222) had their subtype switched to the second-closest centroid. All the switches happened between two adjacent subtypes if they were ordered as LumA‒LumB‒HER2-E‒BL (Supplementary Table 6). The switched subtypes were still very prognostic, and treatment benefit of palbociclib was maintained in all subtypes (data not shown).

## Discussion

**Fig. 4:**
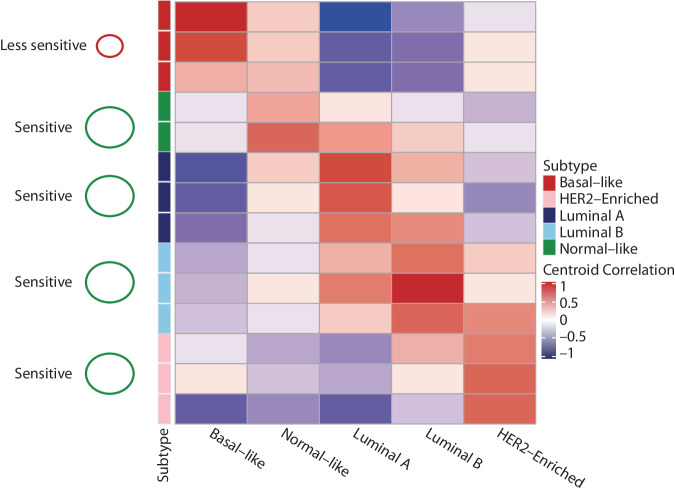
Heatmap of subtype cross-classification. Heatmap of cross-classification of different breast cancer molecular intrinsic subtypes according to PAM50 bioclassifier centroids and their proposed sensitivities to CDK4/6 inhibitors.

This study reports the first analysis with a head-to-head comparison of breast cancer subtyping methods and the clinical association of their differences with survival outcomes in randomized trials. Intrinsic subtyping classification is based on the concept of assigning a tumor to a subtype with the highest similarity to a defined molecular profile, thus representing a spectrum of similarities. This molecular continuum can be clearly seen in Fig. 4, which shows that there is overlap between the different intrinsic subtypes. In this study, we found a considerable lack of agreement (less than 70%) between the different subtyping methods for classifying ER+/HER2− tumors. Using gene-expression data derived from RNAseq in patients with operable breast cancer from PALLET, there was a 69% agreement in assigning molecular subtypes between the RNAseq-AIMS and RNAseq-PAM50.sgMd.TC methods. Using the HTG-derived gene-expression data from patients with advanced breast cancer in PALOMA-2, there was a 54% agreement in assigning molecular subtypes between the HTG-AIMS and ruoProsigna-PAM50 classifying methods. In addition, among the 119 patients in PALOMA-2 in which the two classifying methods were in agreement (clear-defined subtypes), both the HTG-AIMS and ruoProsigna-PAM50 classifying methods were highly prognostic for PFS among the subtypes; however, for the 103 patients in which the two classifying methods did not agree (discord subtypes), the HTG-AIMS and ruoProsigna-PAM50 classifying methods were poorly prognostic for PFS. These findings highlight the limitations in clearly assigning a tumor to a specific molecular subtype and indicate that caution should be taken when evaluating the molecular subtype of tumors.

While there was a lack of agreement in assigning molecular subtypes between methods, the subtyping methods did provide prognostic information for the treatment of palbociclib, consistent with previous studies. A 2020 analysis by Finn et al. presented PFS data by AIMS subtyping in PALOMA-2^17^. The PFS benefit of palbociclib plus letrozole over letrozole plus placebo was greatest among luminal subtypes and reduced in the HER2-like subtype, although observations in the HER2-like subtype were limited by the small sample size (19% of patients). Similar AIMS subtyping results were observed in PALOMA-3, with the majority of patients being assigned a LumA subtype (44.0%), followed by LumB (30.8%), HER2-E (20.9%), NL (2.6%), and BL (1.7%)^18^. A benefit of palbociclib plus fulvestrant versus placebo plus fulvestrant was also observed regardless of the luminal subtype. However, patients with the LumA subtype had longer PFS than patients with the LumB subtype. In the current study, when patients in PALOMA-2 were subtyped by the HTG-AIMS method, palbociclib plus letrozole versus placebo plus letrozole also demonstrated a significant PFS benefit in patients with LumA and LumB subtype. When the PALOMA-2 samples were reanalyzed with the ruoPAM50-Prosigna classifier, results also demonstrated that palbociclib provided a PFS benefit in all patients regardless of the luminal subtype, and additionally similarly benefited the HER2-E subtype; however, the benefit of palbociclib plus letrozole was marginal in patients with the BL subtype. These findings align with those reported in an analysis of 1303 tumor samples from patients with HR+/HER2− disease across the MONALEESA-2, MONALEESA-3, and MONALEESA-7 trials, which demonstrated that the cyclin-dependent kinases 4 and 6 (CDK4/6) inhibitor ribociclib plus endocrine therapy benefited patients with tumors characterized by all molecular subtypes except BL^19^. Of note, in this subtyping analysis of tumor samples across the MONALEESA trials, intrinsic subtyping was not assessed with the HTG-PAM50, RNAseq-PAM50, or ruoProsigna-PAM50 methods. This consistency between studies further suggests the relatively robust utility of intrinsic subtyping of the BL subtype as resistant to CDK4/6 inhibitors and could be considered in future biomarker trial design when evaluating the efficacy of CDK4/6 inhibitors alone or in combination with other experimental drugs in an early breast cancer setting. In addition, the benefit of palbociclib for the HER2-E subtype demonstrated in the PALOMA-2 and PALLET cohorts in this study and in the PALOMA-2 cohort by Finn et al., and the benefit of ribociclib for the HER2-E subtype shown in the MONALEESA trials, suggests that there could be a role for CDK4/6 inhibitors in patients with ER+/HER2+ breast cancer, which is also consistent with preclinical data^20^.

This analysis shows the importance of using validated methods as the determination of intrinsic subtypes varied between methods, and not all tumors had a clearly defined subtype, with some having an intrinsic subtype at the boundary of two subtypes. However, despite the lack of agreement between the methods used the prognostic value of PAM50 subtyping prevailed. AIMS resulted in a better classification of LumA versus LumB but separated LumB and HER2-E poorly. Prosigna PAM50 defined HER2-E clearly but did not provide as clear a distinction between LumA and LumB. In this analysis of intrinsic subtypes, palbociclib plus endocrine therapy should be considered for all patients with ER+/HER2− metastatic breast cancer; the value of alternative treatment for patients with BL tumors warrants future evaluation. A standardized clinical intrinsic subtyping assay and bioinformatics approach such as the PAM50 classifier should be used in clinical practice because discrepancies in gene-expression platforms and algorithms may lead to different results and could misdirect treatment decisions.

## Methods

### Patient population

PALOMA-2 (NCT01740427) was a double-blind, randomized, phase 3 study, in which 666 postmenopausal women with ER+/HER2− advanced breast cancer with no prior treatment for advanced disease were randomly assigned 2:1 to receive palbociclib plus letrozole or placebo plus letrozole^21^. The primary endpoint was investigator-assessed PFS. The details of PALOMA-2 have been previously published^21^; results demonstrated significantly longer PFS with palbociclib plus letrozole compared with placebo plus letrozole.

PALLET was a randomized, multicenter, phase two neoadjuvant trial^22^. Postmenopausal women with unilateral, operable, ER+/HER2− tumors that were ≥2 cm as observed by ultrasound with no evidence of metastatic disease were randomly assigned in a ratio to 3:2:2:2 (A:B:C:D) to 1 of 4 treatment groups. Group A received letrozole alone for 14 weeks; group B received letrozole for 2 weeks followed by palbociclib plus letrozole for 12 weeks, for a total duration of 14 weeks; group C received palbociclib for 2 weeks followed by palbociclib plus letrozole for 12 weeks, for a total duration of 14 weeks; and group D received palbociclib plus letrozole for 14 weeks. The parallel 4-group design allowed the role of each drug in the suppression of the proliferation marker Ki-67 to be evaluated both alone and/or in combination. Ki-67 was centrally assessed. The main results of PALLET have been previously published^22^; briefly, adding palbociclib to letrozole significantly enhanced the suppression of malignant cell proliferation (Ki-67) in people with primary ER+ breast cancer but did not increase the clinical response rate over 14 weeks.

PALOMA-2 was approved by the WCG institutional review board in accordance with the International Council on Harmonization Good Clinical Practice guidelines and the provisions of the Declaration of Helsinki^21^. PALLET was approved by the National Research Ethics Service Committee London—Fulham in accordance with the International Ethical Guidelines for Biomedical Research Involving Human Subjects^22^. For both trials, all patients provided written informed consent and an independent data and safety monitoring committee met every 6 months to review safety data and perform the interim analysis.

### Tissue samples

In PALOMA-2, submission of FFPE tumor samples was mandatory, as previously described^17^. Patients consented to the evaluation of biomarkers associated with sensitivity and/or resistance to palbociclib plus letrozole per the study protocol. In PALLET, core-cut biopsies and trial-specific blood samples were obtained at baseline (after randomization), 2 weeks (before the start of the second drug for groups B and C), and at 14 weeks or at the discontinuation of study therapy (within 48 h of the last dose of study treatment)^22^.

### Gene-expression profiling

In PALOMA-2, gene-expression profiling assays were only performed on samples from patients who had consented to their use. Analyses of gene expression (RNA) were performed with the EdgeSeq Oncology Biomarker Panel (HTG Molecular Diagnostics, Inc), as previously reported^17^. RNA expression levels of 2549 gene targets in FFPE tissues were quantified with targeted capture sequencing. The first section of breast cancer FFPE tissue was stained with hematoxylin and eosin (H&E). The tumor cell content and tissue necrosis were assessed by a board-certified pathologist, and the number of malignant cells as a proportion of all cells (i.e., malignant plus normal cells in the tissue section) was used to estimate tumor content. Acceptance criterion for analysis of tumors was established at >70% of tumor content, and the percentage of necrotic tissue within the total tissue area was used to determine necrosis. The necrosis acceptance criterion for analysis was established at <20% necrosis. If the tumor content was <70% or if necrosis was ≥20%, macrodissection was performed on the tissue sections per standard laboratory processes and manufacturer protocols. Sequencing was performed on an Illumina NextSeq 500 sequencer (Illumina, Inc.). For normalization, probe counts were transformed into log_2_ counts per million. Expression values were quantile normalized. HTG Molecular Diagnostics, Inc., was blinded to patient information and clinical outcomes.

In PALOMA-2, research-use-only (RUO) PAM50 NanoString Breast Cancer Prognostic Gene Signature Assay using the NanoString nCounter Dx Analysis System was validated and implemented at HistoGeneX, Belgium. NanoString confirmed that the kit components and instructions for use were identical between the RUO PAM 50 NanoString Breast Cancer Prognostic Gene Signature Assay kit and investigational-use-only-labeled PAM50 NanoString Breast Cancer Prognostic Gene Signature Assay kit. FFPE breast tumor tissue blocks were submitted to HistoGeneX, and the FFPE tumor blocks were sectioned for ≤10 tissue slides of 5 µm each. A certified pathologist reviewed and evaluated the prepared H&E slides to confirm the area of breast carcinoma and tumor surface area were suitable for PAM50 testing before sample processing. If confirmed that no tumor was present, sample processing was canceled, and results were not reported for that sample. After macrodissection of the tumor area, RNA was extracted, and an elution volume of 30 µL was used for analysis. nCounter gene expression analysis was performed at HistoGeneX in batches of ≤10 samples along with a duplicate control sample. Raw data reporter code count files were transferred to NanoString for analysis using a software module with the same normalization and algorithm used for the investigational use only PAM50 NanoString Breast Cancer Prognostic Gene Signature Assay, which reports the intrinsic subtyping according to the PAM50 gene expression algorithm. In addition, a 300-ng aliquot in a volume of 12 µL was submitted to NanoString for further analysis. NanoString transferred the PAM50 results to HistoGeneX and Pfizer for statistical analyses. Both HistoGeneX and NanoString were blinded to patient information and clinical outcomes.

In PALLET, RNA sequencing (RNAseq) of baseline samples was performed on fresh frozen biopsies for 224 patients (letrozole only, *n* = 77; letrozole plus palbociclib, *n* = 147). Transcriptome RNAseq was performed using total RNA. Strand-specific, poly-A+ RNAseq libraries for sequencing on the Illumina platform were prepared as previously described^23^. Strand-specific, poly-A+ RNAseq libraries for sequencing were prepared with the Illumina platform. At the ligation step, Illumina unique dual barcode adapters (Cat# 20022370) were ligated onto samples. Libraries were amplified in 50-µL reactions containing 150 pmol of P1.1 (5’-AATGATACGGCGACCACCGAGA) and P3 (5’-CAAGCAGAAGACGGCATACGAGA) primer and Kapa HiFi HotStart Library Amplification kit (Cat# kk2612, Roche Sequencing and Life Science). The following PCR conditions were used: incubation at 95 °C for 45 s; followed by 13–15 cycles of 95 °C for 15 s, 60 °C for 30 s, and 72 °C for 1 min; and 1 cycle at 72 °C for 5 min. The amplified libraries were purified with 1.4× AMPure XP beads and eluted into 50 µL of H_2_O. Libraries were quality controlled on a fragment analyzer using a DNA7500 kit (5067–1506, Agilent Technologies), and library yields were determined based on a range of 200–800 bp. Libraries were pooled in equimolar ratios and sequenced on the Illumina platform. Of 427 samples, 34 were sequenced on a HiSeq 2000/2500 instrument to generate 2 × 100-bp reads, and the remaining samples were sequenced on a NovaSeq 6000 instrument using the S4 reagent kit (300 cycles) to generate 2 × 150-bp paired-end reads. An average of 82 million reads per sample were generated. The raw reads of the RNAseq data were aligned to the human genome GRCh38 with gene annotation GENCODE v22 using STAR (v2.5.3a). The read count (i.e., the number of reads mapped to each gene) was produced using HTSeq (v0.12.4). The RNAseq gene expression was evaluated by the upper quartile fragments per kilobase of transcript per million mapped reads, which normalized the read count by dividing it by the gene length and the 75th percentile read count of protein-coding genes for the sample.

Because the EdgeSeq Oncology platform has not been used to profile diverse, large reference tumor sets, and because the PALOMA-2 study only included patients with ER+ disease, the widely used PAM50 classification scheme was not feasible. Instead, the single sample predictor algorithm AIMS (referred to as HTG-AIMS in this paper) used a set of binary rules to compare expression measurements for pairs of genes to classify tumors into intrinsic subtypes for each patient^16,17^. Because only 42 of the 100 binary rules could be applied based on genes in the EdgeSeq Oncology BM panel, classification performance was assessed by downsampling the cancer genome atlas (TCGA) data from genome-wide to the EdgeSeq oncology panel subset. Furthermore, the impact of using 42 rules on the agreement between AIMS and PAM50 is minimal, since the AIMS subtype derived from the 42 rules is highly consistent with those derived from the 100 rules. Using all genes versus EdgeSeq Oncology panel genes only, the agreement between the AIMS subtypes and those classified by PAM50 was 77% vs 76%, respectively^17^. For PALLET, AIMS was applied to the whole transcriptomic data as described and referred to as RNAseq-AIMS^16^.

A summary of the PAM50 algorithm used in PALOMA-2 provided by NanoString is as follows, and results from this algorithm are referred to as ruoProsigna-PAM50 in this paper^24^. The NanoString RUO PAM50 algorithm is a 50-gene signature measuring the gene expression profile of each sample that allows for the classification of breast cancer into four biologically distinct subtypes (LumA, LumB, HER2-E, and BL)^9^. Quality control thresholds are determined using the geometric mean of eight housekeeping genes (HK Geomean), six positive controls, and eight negative controls to ensure RNA quality, and all samples must pass quality-control thresholds for results to be reported. Signal normalization is performed using the eight housekeeping genes. The algorithm was performed in three steps. The first step involves scaling using two sets of scaling factors to bring the housekeeping and reference sample expression values into the scale necessary for the next step. The second step calculates the Pearson correlation between the observed scaled expression for the PAM50 genes and a centroid for each of the four subtypes, resulting in a set of four correlation values for each sample. The final step is to identify the subtype correlation with the greatest value and set that subtype as the subtype call for that sample.

Intrinsic subtypes of the PALOMA-2 cohort were identified based on the HTG data using the PAM50 classifier^9^ with subgroup-specific gene percentile centering (sgPct) as suggested by Zhao et al.^15^. Data were available for 49 of the 50 genes used for the PAM50 classifier. Subtyping results associated with this method are referred to as HTG-PAM50.sgPct in this paper (Supplementary Fig. 1). Each gene in the HTG data was centered on a specific percentile, where the percentile was determined from the RNAseq data of the ER+/HER2− subgroup of the TCGA breast cancer cohort. In the TCGA cohort, we took the percentile of the ER+/HER2− subgroup where the expression value corresponded to the global median expression value of the entire cohort for each gene. Then, in the PALOMA-2 cohort, the TCGA-derived subgroup percentile was assigned to each corresponding gene, and the gene expression was centered by subtracting the value at this percentile. The PAM50 classifier was then applied to the sgPct-centered HTG data to obtain the intrinsic subtypes. Technical calibration was not performed on the HTG data because there was a lack of data performed on both the HTG and microarray platforms. Tumor samples from consenting patients in the PALOMA-2 trial were subtyped using the validated RUO PAM50 assay (ruoProsigna-PAM50); results were compared with published subtype results using AIMS on EdgeSeq Oncology Biomarker Panel (HTG-AIMS; HTG Molecular Diagnostics®, Tucson, AZ, USA; Supplementary Fig. 1)^17^.

In PALLET, PAM50 subtyping was performed on data normalized with subgroup-specific gene centering and microarray-RNAseq calibration, which is labeled as RNAseq-PAM50.sgMd.TC. Because the publicly available PAM50 classifier was developed and validated for breast cancer subtype determination based on microarray data^9^, additional steps should be performed to calibrate the technical bias between RNAseq and microarray platforms when applying the PAM50 classifier to PALLET RNAseq data. RNAseq-PAM50.sgMd.TC includes a two-step calibration: subgroup-specific gene median centering normalization to correct clinical group bias and technical calibration for RNAseq to correct platform bias, as shown in Supplementary Fig. 1. Each gene in the PALLET RNAseq data was centered to the median of the ER+/HER2− subgroup of the TCGA cohort by subtracting the differences between the PALLET median and the TCGA subgroup median from PALLET gene expression^5^. The subgroup-median-normalized RNAseq data were scaled to pretrained microarray-to-RNAseq technical calibration factors to correct the RNAseq bias to microarray before intrinsic subtype classification by the PAM50 classifier^5,9^.

Additional methods included the publicly available PAM50 classifier applied directly on PALLET RNAseq data (RNAseq-PAM50), the publicly available PAM50 classifier with subgroup-specific percentile gene centering (RNAseq-PAM50.sgPct)^15^, and the publicly available PAM50 classifier with subgroup-specific median gene centering (RNAseq-PAM50.sgMd).

We are reporting the NL as was done in the original classifier. Prosigna did not include an NL subtype, therefore no NL was reported. Prosigna assigned NL to LumA. PAM50 and AIMS have NL breast cancer, therefore NL is included in the classifier.

### Statistical analyses

In PALOMA-2, PFS was estimated using the Kaplan–Meier method, hazard ratios were calculated using Cox proportional hazard models, and 1-sided *P* values were calculated by the log-rank test. The agreements between intrinsic subtypes defined by the computational methods were compared by kappa statistics, and percentages were reported as descriptive statistics.

In PALLET, patients with breast cancer with Ki-67 ≤ 2.7% after 14 weeks were classified as achieving complete cell cycle arrest (CCCA), and patients with Ki-67 > 2.7% were classified as not achieving CCCA (non-CCCA), which suggested resistance to treatment^22^. Odds ratios of non-CCCA and 95% CIs were estimated by Fisher’s exact test for each subtype.

### Supplementary information

Supplemental Material

Related Manuscript File

## Data Availability

Upon request, and subject to review, Pfizer will provide the data that support the findings of this study. Subject to certain criteria, conditions, and exceptions. Pfizer may also provide access to the related individual de-identified participant data. See https://www.pfizer.com/science/clinical-trials/trial-data-and-results for more information.

## Code availability

The PAM50 and AIMS codes are publicly available. All steps for the data analyses and processes are described in the methods.

## References

[1] Rugo, H. et al. Endocrine therapy for hormone receptor-positive metastatic breast cancer: American Society of Clinical Oncology guideline. *J. Clin. Oncol.* **34**, 3069–3103 (2016). 27217461 10.1200/JCO.2016.67.1487

[2] Thomssen, C., Balic, M., Harbeck, N. & Gnant, M. St. Gallen/Vienna 2021: a brief summary of the consensus discussion on customizing therapies for women with early breast cancer. *Breast Care (Basel)* **16**, 135–143 (2021). 34002112 10.1159/000516114 PMC8089428

[3] Perou, C. M. et al. Molecular portraits of human breast tumours. *Nature* **406**, 747–752 (2000). 10963602 10.1038/35021093

[4] Sorlie, T. et al. Gene expression patterns of breast carcinomas distinguish tumor subclasses with clinical implications. *Proc. Natl. Acad. Sci. USA* **98**, 10869–10874 (2001). 11553815 10.1073/pnas.191367098 PMC58566

[5] Carey, L. A. et al. Molecular heterogeneity and response to neoadjuvant human epidermal growth factor receptor 2 targeting in CALGB 40601, a randomized phase III trial of paclitaxel plus trastuzumab with or without lapatinib. *J. Clin. Oncol.* **34**, 542–549 (2016). 26527775 10.1200/JCO.2015.62.1268 PMC4980567

[6] Llombart-Cussac, A. et al. HER2-enriched subtype as a predictor of pathological complete response following trastuzumab and lapatinib without chemotherapy in early-stage HER2-positive breast cancer (PAMELA): an open-label, single-group, multicentre, phase 2 trial. *Lancet Oncol.* **18**, 545–554 (2017). 28238593 10.1016/S1470-2045(17)30021-9

[7] Acheampong, T., Kehm, R. D., Terry, M. B., Argov, E. L. & Tehranifar, P. Incidence trends of breast cancer molecular subtypes by age and race/ethnicity in the US from 2010 to 2016. *JAMA Netw. Open* **3**, e2013226 (2020). 32804214 10.1001/jamanetworkopen.2020.13226 PMC7431997

[8] Sestak, I. et al. Comparison of the performance of 6 prognostic signatures for estrogen receptor-positive breast cancer: a secondary analysis of a randomized clinical trial. *JAMA Oncol.* **4**, 545–553 (2018). 29450494 10.1001/jamaoncol.2017.5524 PMC5885222

[9] Parker, J. S. et al. Supervised risk predictor of breast cancer based on intrinsic subtypes. *J. Clin. Oncol.* **27**, 1160–1167 (2009). 19204204 10.1200/JCO.2008.18.1370 PMC2667820

[10] Russnes, H. G., Lingjaerde, O. C., Borresen-Dale, A. L. & Caldas, C. Breast cancer molecular stratification: from intrinsic subtypes to integrative clusters. *Am. J. Pathol.* **187**, 2152–2162 (2017). 28733194 10.1016/j.ajpath.2017.04.022

[11] Nielsen, T. O. et al. A comparison of PAM50 intrinsic subtyping with immunohistochemistry and clinical prognostic factors in tamoxifen-treated estrogen receptor-positive breast cancer. *Clin. Cancer Res.* **16**, 5222–5232 (2010). 20837693 10.1158/1078-0432.CCR-10-1282 PMC2970720

[12] Tibshirani, R., Hastie, T., Narasimhan, B. & Chu, G. Diagnosis of multiple cancer types by shrunken centroids of gene expression. *Proc. Natl. Acad. Sci. USA* **99**, 6567–6572 (2002). 12011421 10.1073/pnas.082099299 PMC124443

[13] Dowsett, M. et al. Comparison of PAM50 risk of recurrence score with oncotype DX and IHC4 for predicting risk of distant recurrence after endocrine therapy. *J. Clin. Oncol.* **31**, 2783–2790 (2013). 23816962 10.1200/JCO.2012.46.1558

[14] Gnant, M. et al. Predicting distant recurrence in receptor-positive breast cancer patients with limited clinicopathological risk: using the PAM50 risk of recurrence score in 1478 postmenopausal patients of the ABCSG-8 trial treated with adjuvant endocrine therapy alone. *Ann. Oncol.* **25**, 339–345 (2014). 24347518 10.1093/annonc/mdt494

[15] Zhao, X., Rodland, E. A., Tibshirani, R. & Plevritis, S. Molecular subtyping for clinically defined breast cancer subgroups. *Breast Cancer Res.* **17**, 29 (2015). 25849221 10.1186/s13058-015-0520-4 PMC4365540

[16] Paquet, E. R. & Hallett, M. T. Absolute assignment of breast cancer intrinsic molecular subtype. *J. Natl. Cancer Inst.* **107**, 357 (2015). 25479802 10.1093/jnci/dju357

[17] Finn, R. S. et al. Biomarker analyses of response to cyclin-dependent kinase 4/6 inhibition and endocrine therapy in women with treatment-naive metastatic breast cancer. *Clin. Cancer Res.* **26**, 110–121 (2020). 31527167 10.1158/1078-0432.CCR-19-0751

[18] Turner, N. C. et al. Cyclin E1 expression and palbociclib efficacy in previously treated hormone receptor-positive metastatic breast cancer. *J. Clin. Oncol.* **37**, 1169–1178 (2019). 30807234 10.1200/JCO.18.00925 PMC6506420

[19] Prat, A. et al. Correlative biomarker analysis of intrinsic subtypes and efficacy across the MONALEESA phase III studies. *J. Clin. Oncol.* **39**, 1458–1467 (2021). 33769862 10.1200/JCO.20.02977 PMC8196091

[20] Finn, R. S. et al. PD 0332991, a selective cyclin D kinase 4/6 inhibitor, preferentially inhibits proliferation of luminal estrogen receptor-positive human breast cancer cell lines in vitro. *Breast Cancer Res.* **11**, R77 (2009). 19874578 10.1186/bcr2419 PMC2790859

[21] Finn, R. S. et al. Palbociclib and letrozole in advanced breast cancer. *N. Engl. J. Med.* **375**, 1925–1936 (2016). 27959613 10.1056/NEJMoa1607303

[22] Johnston, S. et al. Randomized phase II study evaluating palbociclib in addition to letrozole as neoadjuvant therapy in estrogen receptor-positive early breast cancer: PALLET trial. *J. Clin. Oncol.* **37**, 178–189 (2019). 30523750 10.1200/JCO.18.01624

[23] Rokita, J. L. et al. Genomic profiling of childhood tumor patient-derived xenograft models to enable rational clinical trial design. *Cell Rep.* **29**, 1675–1689.e1679 (2019). 31693904 10.1016/j.celrep.2019.09.071 PMC6880934

[24] Wallden, B. et al. Development and verification of the PAM50-based Prosigna breast cancer gene signature assay. *BMC Med. Genom.* **8**, 54 (2015). 10.1186/s12920-015-0129-6 PMC4546262 26297356

