# Supplementary material for: Effect of cross-platform gene-expression, computational methods on breast cancer subtyping in PALOMA-2 and PALLET studies
Source: NPJ Breast Cancer. 2024 Jun 29;10:54. doi: 10.1038/s41523-024-00658-y (PMC11217366; doi:10.1038/s41523-024-00658-y)
Supplement: Supplementary file 1 — Supplemental Material [file 41523_2024_658_MOESM1_ESM.pdf]

Supplementary Information

Supplementary Figure

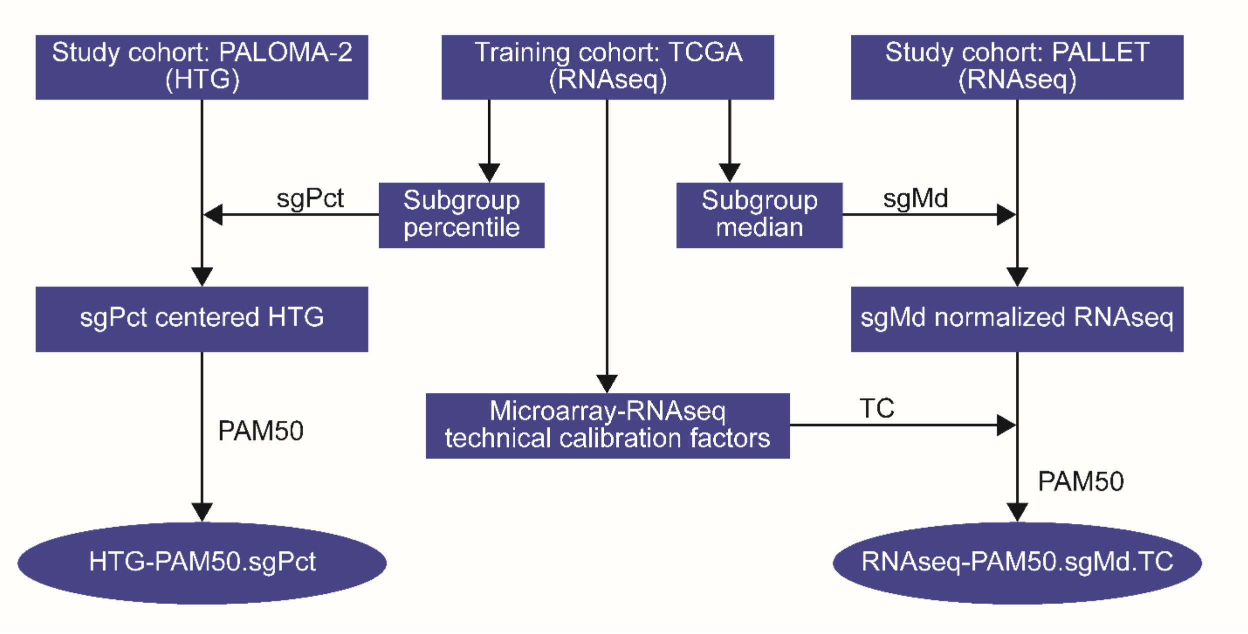

Supplementary Figure 1. Flowchart of PAM50-based intrinsic subtyping with subgroup-specific gene normalization and technical calibration for HTG-EdgeSeq (PALOMA-2) and RNAseq (PALLET). PALOMA-2 PAM50 subtypes were identified by HTG-PAM50.sgPct using the PAM50 classifier with sgPct on the HTG data. PALLET PAM50 subtypes were identified by RNAseq-PAM50.sgMd.TC using the PAM50 classifier with sgMd and microarray-RNAseq technical calibration on the RNAseq data. sgPct, subgroup-specific gene percentile centering; sgMd, subgroup-specific gene median normalization; TC, technical calibration; TCGA, The Cancer Genome Atlas.

Supplementary Tables

Supplementary Table 1. Baseline demographics and disease characteristics of patients in PALOMA-2.

Variable	Overall (N = 666)	Biomarker Data: No (n = 211)	Biomarker Data: Yes (n = 455)	p-value[1]
Age, median (IQR)	62 (54–69)	63 (55–69)	61 (54–68)	0.6
Race, n (%)				0.4
White	516 (81)	157 (77)	359 (82)	
Black	11 (1.7)	3 (1.5)	8 (1.8)	
Asian	95 (15)	37 (18)	58 (13)	
Other	18 (2.8)	6 (3.0)	12 (2.7)	
Treatment, n (%)				>0.9
Letrozole	222 (33)	70 (33)	152 (33)	
Palbociclib + Letrozole	444 (67)	141 (67)	303 (67)	
Number of prior treatment, n (%)				0.3
≥3	68 (16)	26 (20)	42 (15)	
1	206 (49)	65 (50)	141 (49)	
2	144 (34)	39 (30)	105 (36)	
Disease site, n (%)				0.4
Nonvisceral	342 (51)	113 (54)	229 (50)	
Visceral	324 (49)	98 (46)	226 (50)	
Prior hormonal treatment, n (%)				0.4
No	290 (44)	97 (46)	193 (42)	
Yes	376 (56)	114 (54)	262 (58)	
Prior chemotherapy, n (%)	322 (48)	103 (49)	219 (48)	0.9
Chemotherapy type, n (%)				0.5
Adjuvant	236 (73)	80 (78)	156 (71)	
Adjuvant/neoadjuvant	33 (10)	9 (8.7)	24 (11)	
Neoadjuvant	53 (16)	14 (14)	39 (18)	
ECOG PS, n (%)				0.5
0	359 (54)	108 (51)	251 (55)	
1	295 (44)	98 (46)	197 (43)	
2	12 (1.8)	5 (2.4)	7 (1.5)	
Disease-free interval, n (%)				0.066
≤12 months	146 (22)	35 (17)	111 (24)	
>12 months	272 (41)	95 (45)	177 (39)	
De novo metastatic	248 (37)	81 (38)	167 (37)	

ECOG PS, Eastern Cooperative Oncology Group performance status; IQR, interquartile range.

[1]Wilcoxon rank sum test; Fisher's exact test; Pearson's Chi-squared test.

Supplementary Table 2. Subtype counts of PALOMA-2 samples by different HTG subtyping methods.

Method	Normal-like	LumA	LumB	HER2-E	Basal-like
HTG-AIMS	4	229	135	85	2
HTG-PAM50	93	103	99	66	94
HTG-PAM50.sgPct	47	221	127	26	34

Supplementary Table 3. Overlap of subtypes between HTG-PAM50 (rows) and HTG-AIMS (columns) for PALOMA-2 samples.

	HTG-AIMS				
**HTG-PAM50**	**Normal-like**	**LumA**	**LumB**	**HER2-E**	**Basal-like**
Normal-like	3	78	4	8	0
LumA	0	85	13	5	0
LumB	0	27	64	8	0
HER2-E	0	18	34	14	0
Basal-like	1	21	20	50	2

Supplementary Table 4. Overlap of subtypes between HTG-PAM50.sgPct (rows) and HTG-AIMS (columns) for PALOMA-2 samples.

	HTG-AIMS				
**HTG-PAM50.sgPct**	**Normal-like**	**LumA**	**LumB**	**HER2-E**	**Basal-like**
Normal-like	3	28	1	15	0
LumA	1	172	31	17	0
LumB	0	22	89	16	0
HER2-E	0	4	10	12	0
Basal-like	0	3	4	25	2

Supplementary Table 5. Subtype counts of PALLET samples by additional subtyping methods.

Method	LumA	LumB	HER2-E	Basal-like	Normal-like
RNAseq-PAM50	49	64	32	32	47
RNAseq-PAM50.sgPct	84	73	20	13	34
RNAseq-PAM50.sgMd	173	47	0	3	1

Supplementary Table 6. Switched subtype (row) versus original PAM50 subtype (column).

	Original PAM50 Subtype			
Switched subtype	LumA	LumB	HER2-E	Basal-like
LumA	61	17	0	0
LumB	11	93	7	0
HER2-E	0	11	11	2
Basal-like	0	0	2	7

A sample's subtype was switched to the 2nd closest centroid if its correlation was within 0.1 of the closest centroid.
